# Isoflavones Production and Possible Mechanism of Their Exudation in *Genista tinctoria* L. Suspension Culture after Treatment with Vanadium Compounds

**DOI:** 10.3390/molecules23071619

**Published:** 2018-07-03

**Authors:** Milan Skalicky, Jan Kubes, Vaclav Hejnak, Lenka Tumova, Jaroslava Martinkova, Jan Martin, Helena Hnilickova

**Affiliations:** 1 Department of Botany and Plant Physiology, Faculty of Agrobiology, Food and Natural Resources, Czech University of Life Sciences Prague, 165 00 Prague, Czech Republic; hejnak@af.czu.cz (V.H.); martinkova@af.czu.cz (J.M.); hnilickova@af.czu.cz (H.H.); 2 Department of Pharmacognosy, Faculty of Pharmacy, Charles University, 500 02 Hradec Králové, Czech Republic; tumova@faf.cuni.cz (L.T.); martin@faf.cuni.cz (J.M.)

**Keywords:** heavy metals, Dyer’s Greenweed, elicitation, plasma membrane transport

## Abstract

The family *Fabaceae* traditionally serves as a food and herbal remedies source. Certain plants serve for treatment of menopausal symptoms based on a presence of typical secondary metabolites, isoflavones. Beside soybean and clovers, other plants or cultures in vitro can produce these molecules. A cultivation in vitro can be enhanced by elicitation that stimulates metabolites biosynthesis via stress reaction. Vanadium compounds have been already described as potential elicitors, and the aim of this study was to determine the impact of NH_4_VO_3_ and VOSO_4_ solutions on isoflavones production in *Genista tinctoria* L. cell cultures. The significant increase of isoflavones content, such as genistin, genistein, or formononetin, was measured in a nutrient medium or dry mass after NH_4_VO_3_ treatment for 24 or 48 h. The possible transport mechanism of isoflavones release as a result of elicitation was further evaluated. An incubation with different transport inhibitors prior to elicitation took effect on isoflavones content in the medium. However, there was a non-ended result for particular metabolites such as genistein and daidzein, where ATP-binding cassette (ABC) or, alternatively, multidrug and toxin extrusion (MATE) proteins can participate. Possible elicitation by some inhibitors was discussed as a result of their pleiotropic effect. Despite this outcome, the determination of the transport mechanism is an important step for identification of the specific transporter.

## 1. Introduction

*Genista tinctoria* (Dyer’s Greenweed) is a bush from the *Fabaceae* family and traditional utilization in the dying industry provided its species name [1]. This plant also found application as a herbal remedy for body detoxification in folk medicine of the Western Mediterranean and Turkey [2]. With regard to quinolizidine alkaloids occurrence, *G. tinctoria* belongs to poisonous plants too [3]. However, the levels of these metabolites could be different in cultures in vitro [4].

*G. tinctoria* is also a source of various isoflavones, such as other plants from *Fabaceae* family. This specific flavonoid subgroup has different a position of the phenyl group as a result of isoflavone synthase activity [5,6]. It is supposed that plants produce flavones and isoflavones for protection against various stresses, either of abiotic or biotic origin [7]. Simple isoflavones, such as genistein or daidzein, are substrates for further metabolism, and the resulting pterocarpans (pisatin, medicarpin) have significant functions in the plant’s defence [8].

The source of abiotic chemical stress is often the presence of various substances in soil that occur naturally or because of human activity [9]. This is also true in the case of heavy metals such as vanadium. In excess, this metal can have negative effects on plant growth. The level of vanadium in soil is about 150 μg/g on average, but vanadium levels were in the range of 1570 to 3600 μg/g near a former South African mine. Leaf samples from nearby graminoids also had increased amounts of this metal compound. The presence of vanadium negatively affects the overall growth of plants [10], when half maximal effective concentration of vanadium in soil solution ranged from 0.8 to 15 mg/L [11]. An analogy with phosphate molecules is the cause of subsequent inhibition activity of important proteins, such as ATPases, protein kinases, ribonucleases, and phosphatases [12]. Moreover, heavy metals, such as this one, can produce reactive oxygen species that are able to harm cells too [13].

However, stress reaction could be used for intentional production (elicitation) of certain secondary metabolites, and the substance that triggers this process is known as an elicitor [14]. In different studies, various types of vanadium compounds affected the production of other secondary metabolites in various plant cultures in vitro [15,16,17]. Vanadium also had a potential elicitation effect in the case of phenolic metabolites in a suspension culture of *Vigna radiata* [18]. Orthovanadate increased both levels of isoflavones glucosides and phenylalanine ammonia-lyase (PAL) enzyme activity. Orthovanadate affected PAL activity in *Petunia hybrida* too, where an increased level of lignin was recorded [19].

PAL is a key enzyme for flavonoid biosynthesis, which takes place in the cytosol membrane of the endoplasmic reticulum (ER) [20]. Nevertheless, the formation of metabolites can have negative effects on certain processes occurring within cells, so they are stored inside vacuoles or exported out of the cells [21]. A number of mechanisms contribute to this movement, including a suitable form of transportation of substances within cells and plant body. ATP-binding cassette (ABC) transporters are part of the membranes, where hydrolysis of ATP provides energy for transport of molecules. Another way includes multidrug and toxin extrusion (MATE) proteins. The exchange of substrates by these transporters is dependent on the proton/membrane gradient. Various ion pumps (plasma membrane and tonoplast H^+^-ATPase, tonoplast H^+^-PPase) maintain this process. Some substances also utilize vesicle transport. Vesicles with metabolites are budded off from various cellular structures (ER, Golgi apparatus) and subsequently merge with the target membrane [22].

The aim of this research was to determine effect of two vanadium compounds on isoflavone production in the dry matter (DM), and their subsequent exudation to the nutrient medium (NM), of *G. tinctoria* suspension culture. Subsequently, several different inhibitors of transport mechanism were applied to clarify a possible process of secondary metabolites release into the medium.

## 2. Results

### 2.1. The Effect of Vanadium Compounds

The effects of vanadium compounds on the production and exudation of five isoflavones were determined in the suspension culture of *G. tinctoria*. The observed aglycones were genistein, 4′-*O*-methylgenistein (biochanin A); daidzein, 4′-*O*-methyldaidzein (formononetin); and 7-*O*-glucoside of genistein and genistin. The tested solutions had concentrations of 1 or 10 μM of added vanadium compounds (NH_4_VO_3_ or VOSO_4_); cultivation time with elicitors were 24 or 48 h.

**Figure 1:**
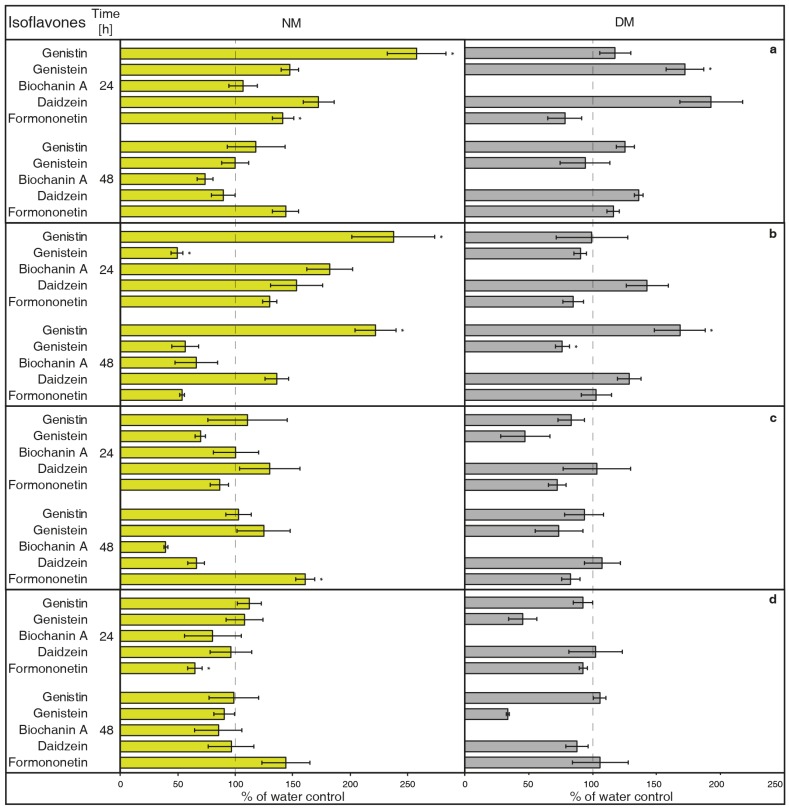
Effects of elicitors on isoflavone content in nutrient medium (NM) and dry matter (DM) after 24 or 48 h. *Genista tinctoria* cell culture was treated by 1 μM (**a**) or 10 μM (**b**) of NH_4_VO_3_ and 1 μM (**c**) or 10 μM (**d**) of VOSO_4_. Bars indicate isoflavone levels in treated samples recalculated to a relative 100% isoflavone content in water control samples. Data are mean of three repeats ± SE; (*) represent significant difference (*p* < 0.05) between tested and water control samples within Tukey’s test.

- NH_4_VO_3_ (1 μM, Figure 1A) has the potential to increase isoflavone biosynthesis and possible metabolites exudation in *G. tinctoria* suspension culture. This compound significantly increased the content of genistin and formononetin in the NM. Genistein and daidzein content also visibly elevated in the NM after 24 h, but their release was statistically insignificant. Samples of DM only had significantly more genistein after 24 h, however, concentration of DM was higher in comparison with the rest of isoflavones. The amount of biochanin A was not traceable in DM and NH_4_VO_3_ (1 μM) took no effect on the release of this isoflavone. Overall, longer cultivation did not cause higher production or exudation of monitored isoflavones.
- NH_4_VO_3_ (10 μM, Figure 1B) also caused a significant increase of genistin content in the NM again after 24 h, but not of formononetin. Moreover, the content of genistein was significantly lower than water control in the NM after 24 h and in the DM after 48 h. In spite of previously used concentration of this elicitor, the content of genistin increased in both NM and DM after 48 h. NH_4_VO_3_ (10 μM) provided no evidence that it is capable of stimulating conceivable production or releasing remaining isoflavones, although daidzein content again was a little higher in both types of samples.
- VOSO_4_ (1 μM, Figure 1C), as the second tested vanadium compound, caused significant release in the case of formononetin content in the NM after 48 h. There was no verifiable difference of other isoflavones content in water control and tested samples after application of this elicitor.
- In a similar way, VOSO_4_ (10 μM, Figure 1D) again caused elevation of formononetin levels in the same samples after 48, but this result was insignificant. Moreover, cultivation for 24 h had a negative effect on this isoflavone presence in the NM. There was a non-significant reduction in genistein content in the DM after 24 h. This result was similar to the genistein value after VOSO_4_ (1 μM) application (Figure 1C); VOSO_4_ did not manifest as a potential elicitor for remaining isoflavones.

Although some results were insignificant, general conclusions suggest that NH_4_VO_3_ has a stronger impact on the production and release of certain isoflavones (genistin). Therefore, NH_4_VO_3_ (1 μM) was subsequently chosen for the study of *G. tinctoria* transport mechanisms. This solution significantly increased content of two isoflavones (genistin, formononetin) in the NM after 24 h. The concentration of genistein and daidzein may also be positively affected; the increase of the former aglycone amount was clearly visible in DM.

### 2.2. The Effects of Transport Mechanism Inhibitors

In order to clarify the possible transport mechanism of the five observed isoflavones mentioned above, the adapted method for *Silybum marianum* was used [23]. Cell cultures were treated with the compounds that are able to suppress transport mechanisms taking place on membranes of various organisms.

**Figure 2:**
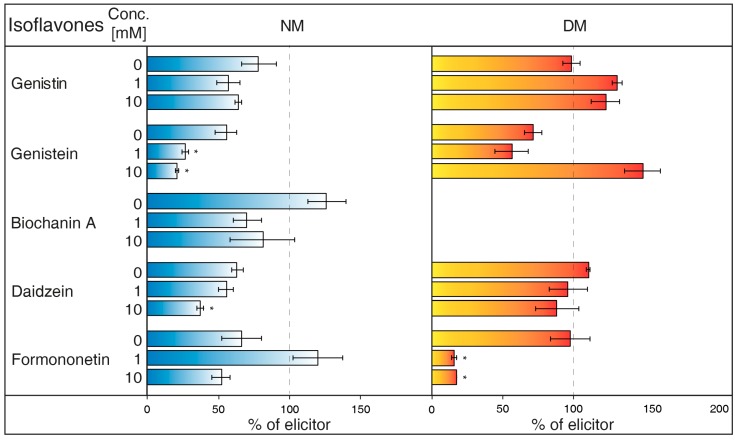
Effects of NH_4_Cl on isoflavone content in nutrient medium (NM) and dry matter (DM) of *Genista tinctoria* cell culture. Zero concentration of inhibitor represents water control. All bar values were recalculated to a relative 100% isoflavone content in samples with NH_4_VO_3_ (1 μM) after 24 h. Data are mean of three repeats ± SE; (*) represent significant difference (*p* < 0.05) between elicited and inhibited or water control samples within Tukey’s test.

**Figure 3:**
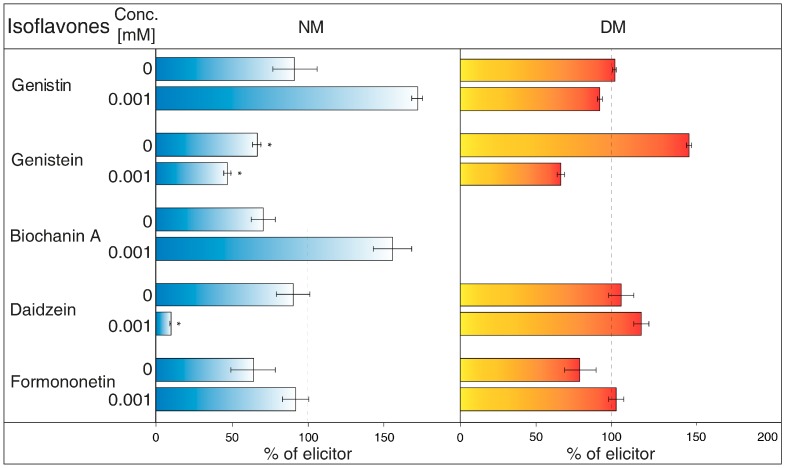
Effects of gramicidin on isoflavone content in nutrient medium (NM) and dry matter (DM) of *Genista tinctoria* cell culture. Zero concentration of inhibitor represents water control. All bar values were recalculated to a relative 100% isoflavone content in samples with NH_4_VO_3_ (1 μM) after 24 h. Data are mean of three repeats ± SE; (*) represent significant difference (*p* < 0.05) between elicited and inhibited or water control samples within Tukey’s test.

**Figure 4:**
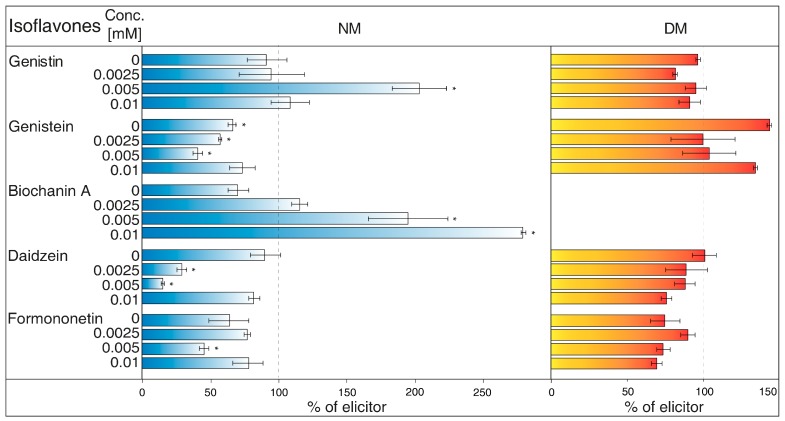
Effects of brefeldin A on isoflavone content in nutrient medium (NM) and dry matter (DM) of *Genista tinctoria* cell culture. Zero concentration of inhibitor represents water control. All bar values were recalculated to a relative 100% isoflavone content in samples with NH_4_VO_3_ (1 μM) after 24 h. Data are mean of three repeats ± SE; (*) represent significant difference (*p* < 0.05) between elicited and inhibited or water control samples within Tukey’s test.

**Figure 5:**
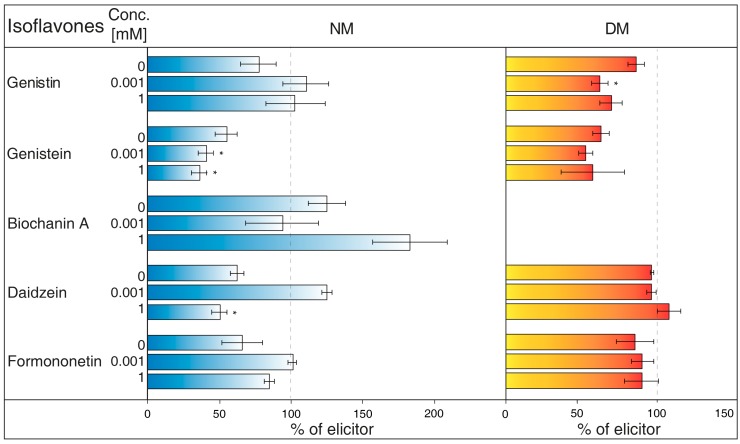
Effects of Na_3_VO_4_ on isoflavone content in nutrient medium (NM) and dry matter (DM) of *Genista tinctoria* cell culture. Zero concentration of inhibitor represents water control. All bar values were recalculated to a relative 100% isoflavone content in samples with NH_4_VO_3_ (1 μM) after 24 h. Data are mean of three repeats ± SE; (*) represent significant difference (*p* < 0.05) between elicited and inhibited or water control samples within Tukey’s test.

**Figure 6:**
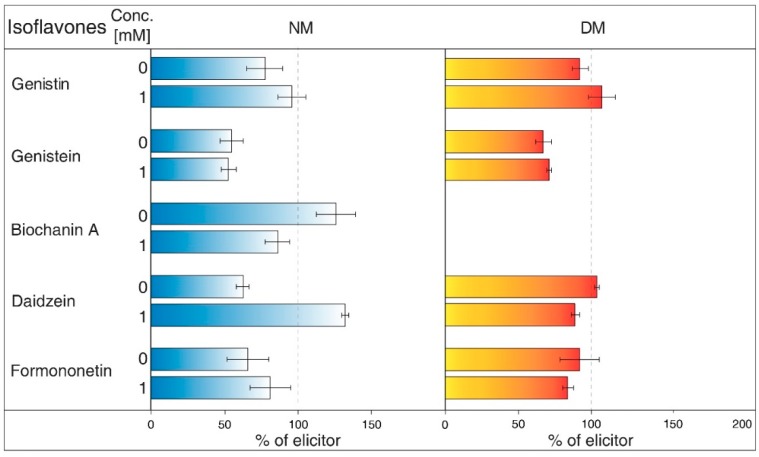
Effects of verapamil on isoflavone content in nutrient medium (NM) and dry matter (DM) of *Genista tinctoria* cell culture. Zero concentration of inhibitor represents water control. All bar values were recalculated to a relative 100% isoflavone content in samples with NH_4_VO_3_ (1 μM) after 24 h. Data are mean of three repeats ± SE.

**Figure 7:**
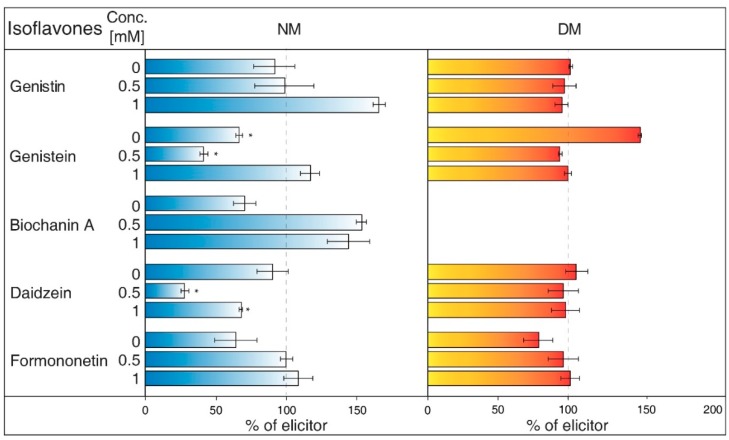
Effects of probenecid on isoflavone content in nutrient medium (NM) and dry matter (DM) of *Genista tinctoria* cell culture. Zero concentration of inhibitor represents water control. All bar values were recalculated to a relative 100% isoflavone content in samples with NH_4_VO_3_ (1 μM) after 24 h. Data are mean of three repeats ± SE; (*) represent significant difference (*p* < 0.05) between elicited and inhibited or water control samples within Tukey’s test.

**Figure 8:**
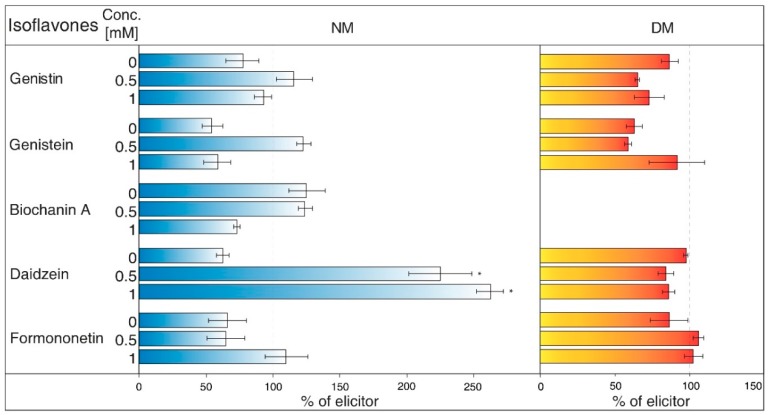
Effects of glibenclamide on isoflavone content in nutrient medium (NM) and dry matter (DM) of *Genista tinctoria* cell culture. Zero concentration of inhibitor represents water control. All bar values were recalculated to a relative 100% isoflavone content in samples with NH_4_VO_3_ (1 μM) after 24 h. Data are mean of three repeats ± SE; (*) represent significant difference (*p* < 0.05) between elicited and inhibited or water control samples within Tukey’s test.

- NH_4_Cl (Figure 2) is ranked between protonophores [24]. It can disrupt the proton gradient and inhibit transport of certain substances, such as nicotine [25]. NH_4_Cl reduced the content of most isoflavones in NM of *G. tinctoria* cell culture, when only genistein (1 mM, 10 mM) and daidzein (10 mM) had significant values in comparison with NH_4_VO_3_ treated samples. In DM, NH_4_Cl had a specific effect, where it significantly decreased formononetin content. Increased genistin and genistein presence in DM was only evident in comparison with water control (not labelled in the figures).
- Gramicidin (Figure 3) is an antibiotic formed by *Bacillus brevis*, which acts as a selective ionophore for cations. Gramicidin disrupted processes associated with potassium ions [26], and eventually transport nicotine in a similar way to NH_4_Cl [25]. The levels of genistein and daidzein significantly decreased in the NM after the application of this inhibitor. Gramicidin, however, did not affect the presence of these two aglycones in DM, as well as other isoflavones in the tested samples. The results also document that the content of genistin and biochanin A was statistically higher in NM after inhibitor application in comparison with the water control (not labelled in the figures).
- Brefeldin A (Figure 4) is a macrolide lactone produced by specific ascomycetes [27]. This inhibitor suppresses the guanine nucleotide exchange factor involved in the vesicular transport of molecules [28]. It also dissolves the Golgi apparatus that contributes on various molecules movement [29]. Brefeldin A suppressed content of some isoflavones content in the NM with significant decrease of genistein, daidzein (2.5 and 5 μM), and formononetin (5 μM). Reduced concentrations of these metabolites could not be verified in NM after treatment with other solutions of this inhibitor. On the other hand, brefeldin A caused a conclusive increase in the levels of genistein (5 μM) and biochanin A (2.5 and 5 μM) in the NM. This inhibitor has no significant effect on any isoflavone content in the DM.
- Na_3_VO_4_ (sodium orthovanadate; Figure 5) is known and used as a plasma membrane (PM) H^+^-ATPase inhibitor. Application of this substance to cell culture of *Eschscholtzia californica* caused gradual alkalisation of surrounding media and a lower rate of excretion of benzophenanthridine alkaloids [30]. In addition, the inhibition caused by Na_3_VO_4_ was discussed for the ABC transporter in *Salmonella typhimurium* [31] and similar transporters in the plants [32]. Na_3_VO_4_ affected isoflavone content according to used concentration. The application of Na_3_VO_4_ (1 mM) strongly reduced the content of genistein and daidzein in the NM, but the less concentrated solution only caused the decrease of genistein concentration. Na_3_VO_4_ had a negative effect on genistin and genistein content in DM, in a case of significant glycoside. Remaining isoflavones also had no greater difference in their amount in these samples.
- Verapamil (Figure 6) inhibits the activity of calcium transport channels, but also has a suppressing effect on multidrug resistance protein 1 (MDR1), a subfamily of ABC proteins. This effect could help with overcoming the tolerance of some drugs caused by P-glycoprotein [33], and *Thalictrum minus* accumulated alkaloid berberine within the cells after this inhibitor application [34]. Verapamil did not cause a significant decrease in any studied isoflavone levels in the NM, but a small reduction for all compounds, except for daidzein, was found. This particular aglycone had a higher concentration than the water control (not labelled in the figures). There was also no verifiable change of isoflavones amount in DM after verapamil application.
- Probenecid (Figure 7) primarily affects the excretion of uric acid in kidneys. This drug can have an effect on the ABC transporters, multidrug resistance-associated protein 1 and 2 subfamily (MRP1 and MRP2), and inhibits the transfer of organic anions [24]. After probenecid treatment, a statistically significant reduction of genistein (0.5 mM) and daidzein (0.5 and 1 mM) was measured in the NM. However, their content did not change significantly in the DM, as well as those of other isoflavones. On the other hand, this inhibitor also positively affects the concentration of genistin (1 mM) and biochanin A in a medium compared with water control samples.
- Glibenclamide (Figure 8) is a drug that acts primarily on ABC proteins in pancreatic B-cells. This drug inhibited the activity of MRP1 in the lung tumour [35], as well as AtMRP5 in *Arabidopsis thaliana* [36]. No isoflavone had a significantly lower content in the NM after glibenclamide application. Nevertheless, there was some reduction of isoflavones (genistein, biochanin A, formononetin). On the contrary, glibenclamide positively affected daidzein content in the NM after administration of both concentrations of the inhibitor, resulting in the opposite effect compared with probenecid. The same effect was found for genistein (0.5 mM) in spite of the water control.

## 3. Discussion

### 3.1. Impact of Vanadium Compounds

Vanadium compounds have already been tested as potential elicitors in the past. Various authors stated that VOSO_4_ increased the production of different secondary metabolites, such as ajmalicine and catharanthine in *Catharanthus roseus* [15]; nicotine in *Nicotiana tabacum*, cv. Virginiana [17]; and ginsenosides in *Panax ginseng* [16]. On the other hand, this elicitor did not enhance content of nicotine in *N. tabacum*, cv. Burley [17]. Some authors also discussed the suppressing effect of this salt on plant growth [17], but the concentration of vanadium solutions was usually higher in these studies. The application of VOSO_4_ (6 mg/L) caused a loss of weight and calcium content in hydroponically grown *Phaseolus vulgaris* (*Fabaceae*) [37], and vanadium was mainly concentrated in the reduced roots [38]. With regard to this research, the suspension culture of *G. tinctoria* was treated with less concentrated solutions of elicitors that should not cause the death of cells

Exact stress mechanism has not been determined, but vanadium and other heavy metals can have direct adverse effect on plants when these elements bind to specific sites instead of regular ions. They can also harm tissues indirectly through self-oxidation or Fenton reaction by production of free radicals [39]. In cells, certain NAD(P)H dependent enzymes are able to make reductions of V^+V^ and form reactive molecules such as H_2_O_2_, and eventually a hydroxyl radical [40].

Plant defence reaction against stress develops as a result of signal cascade triggering. Various messenger molecules carry the signal from receptors with activation of protective mechanisms as the final step [9]. Plant PM contains various proteins, such as protein-like receptors, that can perceive the presence of metals as well as respond to other types of stress factors [41].

As described for human cells [13], vanadium can get into the cytoplasm through the anionic channel (V^+V^) or pass across PM (V^+IV^). Its compounds can then create reactive oxygen species inside cells, which can also react with appropriate receptors. With regard to an inhibition of ATPases of PM by orthovanadate, a subsequent change of membrane potential was proposed as an activation signal for the flavonoid biosynthesis pathway in *Arachis hypogaea* [42]. Higher isoflavone production and exudation was also discussed in another *Fabaceae* plant, *Cicer arietinum*, as a result of different pH values [43]. These changes lead to the flavonoids and related molecules (phenolic acids, lignin) biosynthesis that are dependent on the activity of above-mentioned PAL [18,44]. Heavy metals, such as cadmium and lead, also increase mRNA expression of this enzyme and isoflavones production in *Lupinus luteus* [45]. The mentioned factors could play a role in increase of genistein (Figure 1A; DM).

However, the effects of VOSO_4_ were also dependent on the plant genotype, the monitored tissue, and the concentration of applied solution [17]. This would explain various values of isoflavones content between samples with individual vanadium compounds (Figure 1A,B). The effects of different compounds might vary even at identical concentrations [46]. In a suspension culture of *P. ginseng*, solutions of NH_4_VO_3_ and NaVO_3_ (50 μM) caused the significant increase of ginsenosides in DM, but VOSO_4_ effectivity only had 65% of NaVO_3_. The content of these secondary metabolites was not measured in the medium in their study, while differences in isoflavone levels were determined here after NH_4_VO_3_ and VOSO_4_ treatment (Figure 1A,C).

Beside the character of the elicitor and its quantity, the resulting improvement of secondary metabolites production is dependent on a number of other factors, such as growing conditions and culture type [47]. With regard to genistin (Figure 1B), in summary of secondary metabolites production research, some elicitors also caused a higher quantity of substances in both cells and the medium of certain cultures [48]. In the case of other plants, their metabolites were exported out, but the dry matter content remained unchanged.

The production of isoflavones in *G. tinctoria* in vitro cultures was also compared with that in wild plants [49]. Upon establishing suitable conditions, the in vitro cultures produced more secondary metabolites. The age of the culture is also one factor for biosynthesis of secondary metabolites and young calluses were used in the above-mentioned study. On the other hand, cell cultures of *G. tinctoria*, treated with vanadium compounds or used in subsequent transport experiments, were subcultures from older calluses. Beside the overall results, NH_4_VO_3_ manifested as a promising elicitor for isoflavones production in suspension culture of *Genista tinctoria.* This could be used in further isoflavones metabolism research, while utilization for medicine is questionable so far. However, other authors [16,46] did not work with potential vanadium contamination of secondary metabolites. Some vanadium compounds also had beneficial effect against diabetes [12].

### 3.2. Transport of Isoflavones across Membranes

The ordinary research of metabolite transport mechanism works with isolated PM [50] or tonoplast vesicles [24] together with a known amount of a tested metabolite. These experiments determine the metabolite uptake in the presence of some molecule with the potential to affect the transport process. In contrast, a similar study was conducted [23], in which elicitors stimulated the natural production and exudation of metabolites. Moreover, to bring new light to inhibition effect evaluation, the isoflavone content in the NM of *G. tinctoria* was determined, together with the DM.

Natural substances may be transported in different ways such as above-mentioned ABC transporters, MATE proteins [32,51], and vesicular transport [52]. Various mechanisms were identified with regard to the selected plant, examined membrane (plasma or vacuolar), or the selected metabolite.

In the case of some samples, where inhibitors produced by microorganisms (*Bacillus brevis*, *Eupenicillium brefeldianum*) were used (gramicidin, brefeldin A), a noticeable increase of genistin, and particularly biochanin A, in the NM was recorded (Figure 4 and Figure 5), despite differences in the structure of inhibitor molecules. This could be explained by classification of isoflavones between phytoalexins. They play a significant role in the case of the plant’s exposure to abiotic or biotic stress and this group included a number of different compounds, such as pisatin from *Pisum sativum* or medicarpin from *Medicago truncatula* [53]. These metabolites have a vital part in the interaction between the plant and the microbial pathogen. The inhibitors used could act as biotic elicitors that stimulate biosynthesis.

Plants from the *Fabaceae* family have created symbiosis with nitrification bacteria and form nodules on their roots as a result. Aside from a protective function, the ability of isoflavones and other flavonoids to attract nitrification bacteria is under consideration. The participation of ABC transporters in the transport of isoflavones through PM was found in the roots of *Glycine max*, although some of the used ABC inhibitors did not affect this process. However, possible uptake could be dependent on another subgroup, classified as ABCG or pleiotropic drug resistance (PDR) proteins [50]. In *G. tinctoria*, it was determined that Na_3_VO_4_ (1 mM) solution also caused a strong decrease of genistein and daidzein levels in NM (Figure 5). Moreover, substances such as verapamil suppressing the activity of multidrug resistance protein (MRP) (ABCB) or probenecid, active against MRP (ABCC), may not have a noticeable impact in *G. max*, as in case of *G. tinctoria* (Figure 6). Probenecid (0.5 mM) in the NM only decreased the content of genistein and daidzein; the amount of other isoflavones was not affected (Figure 7).

Another outcome was discussed in a paper, where flavanone naringenin was applied to the root cap of *A. thaliana* [54]. The movement of this metabolite was mostly inhibited in plant samples treated with glibenclamide or glutathione in various redox states. Glutathione, specifically in connection with glutathione S transferase, tends to be associated with the transport dependent on MRP proteins [55]. In spite of the mentioned experiment with *G. max*, Na_3_VO_4_ did not significantly suppress the movement of naringenin. Moreover, the treatment of cell suspension of *G. tinctoria* with the glibenclamide brought about an insignificant decrease of some selected isoflavones (Figure 8), and involvement of the MRP subgroup could be excluded.

The participation of PDR transporters was supposed in the case of *M. truncatula* hairy-root culture, where MtABCG10 was identified. To clarify this protein importance, its gene was silenced and then the amount of medicarpin precursors, such as daidzein, formononetin, and vestitonin, decreased. The plant culture was also more susceptible to the fungal pathogen *Fusarium oxysporum*, indicating the role of these substances as phytoalexins [56]. Another PDR transporter, AtABCG29, was identified in *A. thaliana*, and is responsible for the transmission of monolignol p-coumarylalcohol through PM [57]. This metabolite production is also PAL dependent and it is a component of lignin. It was discussed that transmission of these phenolic aglycones primarily took place on PM, while their glycosides were transported more through tonoplast [58]. The authors referred that various forms of molecules require different transporters for their transmission and structure contributes to the placement of metabolites.

However, transport of the individual isoflavones may be affected by the presence of other similar metabolites. The uptake of genistein into *G. max* PM vesicles was decreased in the presence of other aglycones, particularly daidzein, formononetin, and biochanin A. Therefore, the same type of transporter was hypothesised for these molecules. With regard to glycosides, only genistin had a stronger effect on uptake inhibition in comparison with 7-*O*-glucosides of other flavonoids [50]. In the case of *G. tinctoria,* an export of several isoflavones was observed at the same time; their possible mutual effect on transport cannot be excluded. Abundance of metabolite inside cells caused by the inhibitors could cause further transformation of individual substances (conjugation, methylation, acylation) [59]). Moreover, other isoflavonoids that were not measured are present in *G. tinctoria*. This could then explain some large differences between the measured results.

On the other hand, studies focusing on a tonoplast transport of flavonoids brought more examples of MATE proteins and proton pumps participation within this membrane. The inhibitors of the proton gradient disrupted the uptake of glycoside saponarin in *Hordeum vulgare*, while the ABC proteins mediated flavonoid transport in *A. thaliana* [32]. Saponarin is not a natural metabolite of *A. thaliana*; the participation of a transporter of different mechanisms was discussed with regard to its character as a remover of toxic substances. In *M. truncatula*, more proteins were later identified, such as MtMATE1, which contributed to the transport of flavan-3-ols [51] or MtMATE2 transporting some acylated flavonoids [60]. The vacuolar uptake of daidzin in this plant was more sensitive to inhibitors of the ABC proteins, but the specific transporter was not identified here [51]. Some of the ABC transporters on the tonoplast first identified were ZmMRP3 for anthocyanins in *Zea mays* [61] and VvABCC1 in *Vitis vinifera* [62].

Despite the tonoplast, participation of MATE proteins on flavonoid transport across PM has not yet been reported. In the case of *Vaccinium corymbosum*, several VcMATEs were identified as possible transporters due to their similarity with some known transporters from this family [63]. Therefore, decreased genistein and daidzein content in the NM could indicate involvement of MATE protein (Figure 2). The gramicidin decreased the content of genistein and daidzein, but the levels of these isoflavones did not change in DM (Figure 3).

In the vesicles prepared from the xylem of various woody plants (*Populus sieboldii*, *Pinus densiflora*), the transport of coniferin (coniferyl alcohol glucoside) particularly occurred on the tonoplast and possibly on the ER membrane [64]. The uptake of this metabolite was dependent on the presence of MgATP, while its aglycone was minimally transported under the same conditions. Unlike the transport in *A. thaliana*, bafilomycin A1 inhibited this process in hybrid poplar trees (*Populus sieboldii* × *Populus grandidentata*), because this inhibitor affects the proton gradient through vacuolar ATPase.

In the above mentioned experiments, Na_3_VO_4_ was primarily applied as the inhibitor of ABC proteins. However, older studies used this compound against other enzymes with ATPase activity. In animals, vanadate affected some pumps, such as the (Na^+^/K^+^)-ATPase [65] and (H^+^/K^+^)-ATPase. The inhibition of ATPase was subsequently also shown on the PM in *Beta vulgaris* and *Z. mays* [66]. Aside from orthovanadate, vanadyl sulphate was also able to inhibit (Na^+^/K^+^)-ATPase [67], as was sodium metavanadate [65]. Another compound, ammonium metavanadate, then disrupted the activity of the electrogenic ATPase on the peribacteriod membrane in *G. max* [68]. The inhibition effect of metavanadate and vanadyl on these enzymes was not studied in plant cultures such as *P. ginseng* and *N. tabacum* during elicitation.

In contrast, the effect of orthovanadate as H^+^-ATPase inhibitor on PM that could act as the elicitor by this mechanism was also discussed [43]. A treatment of *C. arietinum* with vanadate (50 μM, 100 μM) caused an increased production and release of pterocarpans (maackiain, medicarpin) and isoflavones (biochanin A, formononetin) from the roots into the medium. The authors proposed a hypothesis that the change of pH of the external environment to more alkaline, as a result of protein pump inhibition, had a positive effect on the elicitation. There was no reduction of secondary metabolites in the medium with increased concentrations of vanadate. The application of NH_4_VO_3_ and VOSO_4_ could cause elicitation in the cell culture of *G. tinctoria* without affecting the release of isoflavones, while their potential effect on ATPase may not have manifested at the concentrations used.

Another aspect of flavonoids movement, particularly anthocyanin, was clarified by describing of transport vesicles in line with occurrence of specific bodies in plant cells [69]. The cyanoplasts and anthocyanoplasts had high levels of these secondary metabolites. It was stated that vesicles contain precursors of proanthocyanidins, which could have been formed by splitting from ER, where the metabolites were initially transported from the cytosol membrane. Protein GFS9 (green fluorescent seed) present in the Golgi apparatus of *A. thaliana* was discussed as one of the vesicular transport factors for vacuolar uptake of flavonoids. Mutants with the *gfs9* gene had a different colour in comparison with wild-type plants and were equated to mutant *tt9* [70].

In a hairy-root culture of *Ophiorrhiza pumila*, an application of brefeldin A caused a release of alkaloid camptothecin into the extracellular space. According to Zhao and Dixon [51], this metabolite is also formed on ER and stored in the vacuole with potential passive transport across PM. Brefeldin A suppressed camptothecin uptake into the vacuole and subsequently increased metabolite in the medium.

Therefore, the disruption of isoflavones exudation by vesicles would manifest as their content increased in the DM. The possibility of passive transport of flavonoids was not extensively researched [71]. However, vesicular transport could participate in the culture of *Silybum marianum* [23], where brefeldin A decreased content of flavonoids in the medium. The application of brefeldin A could have affected the NM concentration of genistein, daidzein, and formononetin (Figure 4) within used cell culture. Overall, it was not possible to definitively determine whether the studied isoflavones are transported by vesicles.

## 4. Materials and Methods

### 4.1. In Vitro Culture Preparation

The Agricultural Faculty of the Mendel University in Brno (CZ) provided the seeds for the plant material cultivation. Manipulation of plant material and all experiments took place in a disinfected box with a laminar flow. The seedlings of *G. tinctoria* were cultivated on a Schenk and Hildebrandt medium [72] with agar under stable conditions (24 °C, 16-h light and 8-h dark regime) under a ColorLux Plus bulb (NARVA Lichtquellen GmbH, Brand-Erbisdorf, Germany) with irradiance of 81.51 μW m^−2^.

Callus cultures were prepared by cutting the stem of seedlings to smaller parts that were placed onto paper bridges immersed in a Schenk and Hildebrandt medium. The medium contained 2,4-D (2.2621 μM; SERVA Electrophoresis GmbH, Heidelberg, Germany) and kinetin (0.4646 μM; SERVA Electrophoresis GmbH, Heidelberg, Germany) as growth hormones that support the formation and development of unorganized tissue. The formed callus cultures were subsequently cultivated in closed Erlenmeyer flasks (250 mL) under the same conditions, and subcultured every four weeks until the tissue became homogenous and sufficiently grown.

A suspension culture was then prepared from this tissue by transferring the plant inoculum (approximately 5 g FW) into a liquid Schenk and Hildebrandt medium with same concentration of used growth hormones. The callus mass was carefully disrupted to smaller cell clusters that were cultivated in Erlenmeyer flasks under the same conditions again. An aeration and prevention of cell sedimentation was achieved by placing the flasks into a multi-flask shaker (115 rotations per min; VKS 75, Edmund Buhler GmbH, Hechingen, Germany). The subculturing of the suspension culture took place every 14 days, when approximately 15 mL of thick suspension was transferred into 20 mL of fresh Schenk and Hildebrandt medium.

### 4.2. Vanadium Treatment

The effects of two vanadium compounds were tested in three-day-old suspension culture of *G. tinctoria*. This culture was treated with 1 mL of ammonium metavanadate (NH_4_VO_3_; Lachema, Brno, CZ, USA) or vanadyl sulfate (VOSO_4_; Honeywell Riedel de Haën, Seelze, Germany) and the concentration of the elicitors was 1 and 10 μM. At the same time, cultures with only 1 mL of distilled water were prepared as a control group.

Flasks with the tested sample suspensions were returned to the shaker and cultivated under the same conditions for 24 and 48 h. Individual samples were later removed and separated at normal pressures through a filter paper. The separated NM was immediately used for extraction of isoflavones, or these liquid samples were frozen and kept refrigerated for no more than one week. Cellular sediment on the filtration paper was carefully rinsed and dried at laboratory temperature. Samples of DM were stored in a dark, dry place until their extraction.

### 4.3. Transport Mechanism Inhibitors Treatment

The result of elicitation with vanadium compounds indicated that the NH_4_VO_3_ (1 μM) solution caused an increase of certain isoflavones level in the NM after 24 h. Several compounds affecting various transport mechanisms were then applied as possible inhibitors of these metabolites exudation. A three-day-old suspension culture was used again and 30 μL of the inhibitors NH_4_Cl (PENTA, Prague, CZ), gramicidin, brefeldin A, Na_3_VO_4_, verapamil (Sigma-Aldrich^®^, Schnelldorf, Germany), probenecid (MSD, Prague, CZ, USA), and glibenclamide (The Roche Group, Basel, Switzerland) from the stock solution, prepared according to Zhao [22], was added.

The cultures were incubated with the inhibitors for 1 h and once again with 1 mL of NH_4_VO_3_ (1 μM) and cultivated for 24 h in accordance with the above conditions for the suspension culture. At the same time, samples containing only 1 mL of the elicitor or 1 mL of distilled water without inhibitors (water control) were prepared by the same method. All samples were filtrated after 24 h and processed as before (Section 4.2). The solvents had a statistically insignificant effect on the level of isoflavones in the DM or the NM.

### 4.4. Extracts Preparation and HPLC Analysis

*Genista tinctoria* extracts and HPLC analysis was done in line with the methodology of the Department of Pharmacognosy. The specific volume of the nutrient media was reduced in the Laborota 4010 Rotary Vacuum Evaporator (Heidolph Instruments, Schwabach, Germany) and the evaporated residue was dissolved in 10 mL of methanol 80%. This solution was placed into a measuring flask (10 mL) and filled up to the mark with the same solvent. The content of the flask was transferred through a syringe with a 0.45-μm microfilter (VWR International, Radnor, PA, USA) into a vial for HPLC analysis.

Dried sediment from the suspension culture was carefully crushed into powder in a mortar, and 0.30 to 0.50 g of DM was then placed in a laboratory boiling flask. The dry cells were extracted by 10 mL of methanol 80% on a water bath (75 °C) under a Liebig cooler for 10 min.

**Figure 9:**
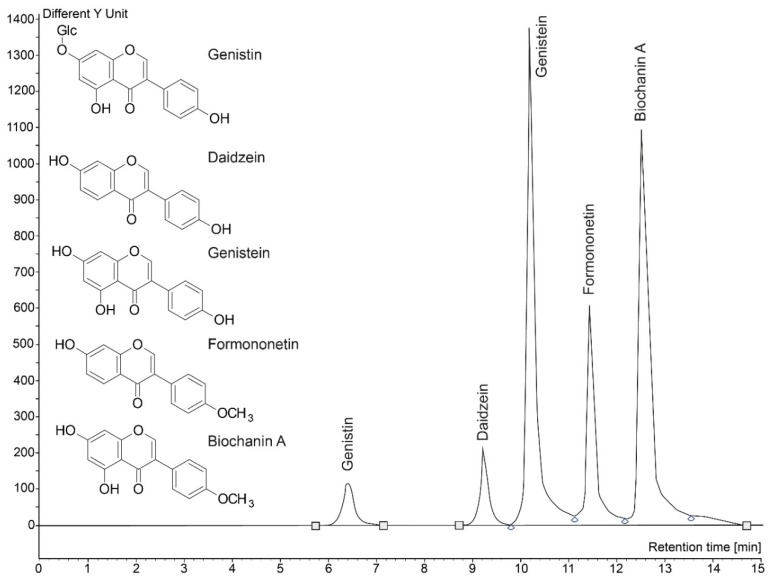
HPLC chromatogram of monitored isoflavones standards and their chemical formula.

The mixture was cooled and filtered through cotton; the liquid component was transferred into a measuring flask (20 mL). DM from the cotton was placed back into the boiling flask, and the extraction process was repeated under the same conditions. The liquid part of the second extraction was mixed with the first and the resulting solution was also placed in to a vial for HPLC analysis using a 0.45-μm microfilter. The isoflavones content in the NM and DM was established using HPLC. Individual isoflavone standards (Sigma-Aldrich, Schnelldorf, Germany; Figure 9), the chromatography set (JASCO International, Tokyo, Japan), and the analysis assay follow used methodology [73].

### 4.5. Statistical Analysis

The values of isoflavones content in control samples in the case of Figure 1, and elicited samples in the cases of Figure 2, Figure 3, Figure 4, Figure 5, Figure 6, Figure 7 and Figure 8, were taken as 100% and they are given in tables (Supplemental Information, Tables S1 and S2). The results of other samples were related to this value. Two subsequent subcultures of suspension culture were used for potential transport mechanism determination. Each tested group was only compared with isoflavone content in corresponding subculture. A mixed-model procedure, with a repeated statement for each parameter, was used to analyse the data set. Data from each measurement was tested separately. Tukey’s test (*p* < 0.05) was used to determine significant differences. All statistical tests presented in this study were performed using a Statistica 13 (StatSoft Inc., Tulsa, OK, USA) software package.

## 5. Conclusions

In summary, this study presented ammonium metavanadate as a potential elicitor of several isoflavones in *G. tinctoria*, because higher production and release of these metabolites has been found. Beside other well-known legumes, this one also represents a possible alternative model source of isoflavones for plant physiology research, or potentially herbal medicine. The activity of important biosynthesis enzymes or stress signal molecules, such as jasmonic acid, was not examined in vanadium treated *G. tinctoria* suspension culture, in contrast to previous studies [46]. The exact clarification of the elicitor mechanism in this cell culture could thus be considered as a challenge for future research. Monitoring the release of isoflavones had less evident results, because more types of inhibitors affected genistein or daidzein transport, similar to the mentioned daidzin in *M. truncatula* [51]. There is also a possibility that a specific inhibitor would show more significant results, confirming the hypothetical participation of protein from the ABCG subfamily. Naturally, plant secondary metabolites transporters are not identical to proteins inhibited by used drugs. However, other plant model systems also explained a variability of inhibitors efficacy and other different compounds can be tested. Unlike *G. max*, *C. arietinum*, or *M. truncatula*, there is no described genome of *G. tinctoria* yet and the determination of the specific transporter protein could be more difficult. Therefore, further experiments dedicated to the transport mechanism are needed, such as the determination of isoflavones movement in the intact plant or across the tonoplast.

## Supplementary Materials

The following are available online at http://www.mdpi.com/1420-3049/23/7/1619/s1. Table S1: The content of isoflavones in nutrient medium (mg/100 mL) and dry matter (mg/g) of *Genista tinctoria* after application of distilled water for 24 or 48 h. Table S2: The content of isoflavones in nutrient medium (mg/100 mL) and dry matter (mg/g) of *Genista tinctoria* after application of NH_4_VO_3_ (10 μM) alone for 24 h. The values were used for a comparison with specific inhibitor in one passage of cell culture.
