# Supplementary material for: Isoflavones Production and Possible Mechanism of Their Exudation in Genista tinctoria L. Suspension Culture after Treatment with Vanadium Compounds
Source: Molecules. 2018 Jul 3;23(7):1619. doi: 10.3390/molecules23071619 (PMC6099964; doi:10.3390/molecules23071619)
Supplement: Supplementary file 1 [file molecules-23-01619-s001.zip › Table S1.pdf]

molecules

MDPI

Table S1. The content of isoflavones in nutrient medium (mg/100 mL) and dry matter (mg/g) of *Genista tinctoria* after application of distilled water for 24 or 48 h.

	Cultivation time [h]	Genistin	Genistein	Biochanin A	Daidzein	Formononetin
Nutrient medium	24	2.9036 ± 0.4115	1.0253 ± 0.1508	0.4229 ± 0.1441	0.5570 ± 0.0407	2.3079 ± 0.1224
	48	2.4361 ± 0.7303	0.8545 ± 0.1806	0.8688 ± 0.1171	0.8816 ± 0.1302	2.1890 ± 0.2681
Dry matter	24	46.4030 ± 10.6300	2.2117 ± 0.5144	-	0.3663 ± 0.0633	0.5323 ± 0.2065
	48	45.6143 ± 4.62356	2.6477 ± 0.2492	-	0.4057 ± 0.0442	0.3770 ± 0.0745

Data are mean ± SE of three repeats.
