# Supplementary material for: Isoflavones Production and Possible Mechanism of Their Exudation in Genista tinctoria L. Suspension Culture after Treatment with Vanadium Compounds
Source: Molecules. 2018 Jul 3;23(7):1619. doi: 10.3390/molecules23071619 (PMC6099964; doi:10.3390/molecules23071619)
Supplement: Supplementary file 1 [file molecules-23-01619-s001.zip › Table S2.pdf]

molecules

MDPI

Table 2. The content of isoflavones in nutrient medium (mg/100 mL) and dry matter (mg/g) of *Genista tinctoria* after application of NH_4VO_3 (10 µM) alone for 24 hours. The values were used for a comparison with specific inhibitor in one passage of cell culture.

	Inhibitor	Genistin	Genistein	Biochanin A	Daidzein	Formononetin
Nutrient medium	NH_4Cl Na_3VO_4 Verapamil Glibenclamide	3.9106 ± 0.5044	2.2895 ± 0.6905	0.6720 ± 0.2498	0.8022 ± 0.0352	3.6870 ± 0.2248
	Gramicidin Brefeldin A Probenecid	3.4416 ± 0.7951	1.3079 ± 0.1854	0.3431 ± 0.0238	1.9948 ± 0.1251	3.4535 ± 0.4204
Dry matter	NH_4Cl Na_3VO_4 Verapamil Glibenclamide	76.5500 ± 7.7996	1.2230 ± 0.1548	-	0.3877 ± 0.0212	0.2997 ± 0.0153
	Gramicidin Brefeldin A Probenecid	69.7713 ± 5.8396	0.6677 ± 0.1420	-	0.3310 ± 0.0098	0.3820 ± 0.0286

Data are mean ± SE of three repeats.
